# Comparison of prognoses according to non‐positive and positive spectrin *α*II expression detected immunohistochemically in epithelial ovarian carcinoma: a retrospective study.

**DOI:** 10.1002/cam4.683

**Published:** 2016-03-19

**Authors:** Osamu Maeda, Tomoko Miyata‐Takata, Kiyosumi Shibata, Hiroaki Kajiyama, Mika Mizuno, Koji Tamakoshi, Yoshie Shimoyama, Shigeo Nakamura, Fumitaka Kikkawa

**Affiliations:** ^1^ Department of Obstetrics and GynecologyNagoya University Graduate School of MedicineTsurumai‐cho 65, Showa‐kuNagoya466‐8550Japan; ^2^ Department of PathologyFaculty of MedicineOkayama UniversityOkayamaJapan; ^3^ Department of GynecologyAichi Cancer Center HospitalNagoyaJapan; ^4^ Department of HealthNagoya University Graduate School of MedicineNagoyaJapan; ^5^ Department of Pathology and Laboratory MedicineNagoya University of HospitalNagoyaJapan

**Keywords:** Anticancer drug resistance, biomarker, immunohistochemistry, ovarian carcinoma, prognosis, spectrin *α*II

## Abstract

Anticancer drug sensitivity affects prognosis in ovarian carcinoma. Previously, we purified spectrin *α*II and *β*II tetramers from cisplatin‐resistant ovarian serous adenocarcinoma cells and demonstrated that they contribute to platinum anticancer drug resistance. In this clinical study, we focused on the role of spectrin *α*II expression. It is our objective to demonstrate the potential of spectrin *α*II expression as a useful predictor of anticancer drug resistance and postoperative prognosis in epithelial ovarian carcinoma. Spectrin *α*II expression in the ovarian adenocarcinoma surgical specimens of 193 patients was examined by immunohistochemical staining. Staining strength was scored 3+, regarded as positive expression, and 2+, 1+, and 0, regarded as non‐positive expression. Prognoses obtained from clinical records were evaluated by statistical analysis. In the 193 cases studied, positive spectrin *α*II expression was associated with worse overall survival when compared with non‐positive expression (*P *< 0.001 by log‐rank test), and spectrin *α*II expression was identified as an independent predictive factor of overall survival (hazard ratio[HR]: 3.77, 95% confidence interval[CI]: 1.77–8.00; *P *< 0.001 by multivariate Cox's proportional hazards model). In the study about progression‐free survival, spectrin *α*II expression was not associated with prognoses. However, similar results as overall survival were obtained for survival after recurrence of the 92 recurrent cases (*P *= 0.0051 by log‐rank test, HR: 4.49, 95% CI: 2.06–9.79; *P *< 0.001 by multivariate Cox's proportional hazards model). In a detailed overall survival study of 66 serous adenocarcinoma patients and 127 nonserous adenocarcinoma patients, similar results were also obtained. Spectrin *α*II expression is a useful predictor of anticancer drug resistance and postoperative prognosis in epithelial ovarian carcinoma..

## Introduction

The number of cases of ovarian cancer is increasing 1, so it is important to discover more effective treatments. Paclitaxel and carboplatin are administered as a standard combination chemotherapy regimen 2, 3. Many other anticancer drugs have been developed to treat ovarian cancer including gemcitabin, liposomal doxorubicin, and topotecan. Use of these drugs gives us many therapeutic options 4. Currently, molecular target‐based drugs such as bevacizumab that inhibit vascular endothelial growth factor are introduced in usual clinical treatment 5, 6. Although paclitaxel and carboplatin are administered to all ovarian cancer patients routinely, this therapy does not always result in a favorable prognosis due to anticancer drug resistance.

We have continued to study drug resistance in ovarian carcinoma 7, 8, 9. In a previous paper, we reported purification of two proteins in the 300 kDa range from cisplatin‐resistant cells by affinity chromatography with cisplatin‐exposed Glutathione Sepharose 4B. The purified proteins were identified as spectrin *α*II and *β*II 10, 11 by peptide mass mapping analysis. These proteins were expressed more strongly in resistant cells than in sensitive cells. We demonstrated that reduction of spectrin *α*II expression increased sensitivity for platinum drugs. In a clinical study, we showed that spectrin *β*II expression may increase after anticancer drug treatment. Furthermore, patients with detectable residual tumors at the time of surgery whose tumor specimens stained strongly for spectrin *β*II had shorter CA125 progression‐free survival periods than patients whose tumor specimens stained weakly. Thus, we demonstrated that spectrin *α*II and *β*II tetramers contribute to platinum anticancer drug resistance in ovarian serous adenocarcinoma 12.

It is our general objective to elucidate the novel mechanisms of drug resistance in ovarian cancer for use in clinical therapy to enable selection of anticancer drugs that are effective for refractory patients. Patient prognosis can be improved if we are able to determine on a case‐by‐case basis before chemotherapy treatment is begun which chemotherapy regimen will overcome the resistance mechanism and be most effective. In this study for clinical application, it is our objective to demonstrate the potential of spectrin *α*II expression as a useful predictor of anticancer drug resistance and postoperative prognosis in epithelial ovarian carcinoma.

## Materials and Methods

### Patients and tissues

This study is a retrospective study, which shows comparison of prognoses according to spectrin *α*II expression detected immunohistochemically in epithelial ovarian carcinoma. In this paper, series of initial treatments means the successive chemotherapies and surgeries initially undergone by a patient without interval. Treatment for recurrence is excluded from the series of initial treatments. When a second debulking surgery was performed after interval due to recurrence, it was excluded from the series of initial treatments. In this case, the count began with the day the first surgery was performed. A group of physicians conferred in each case to determine whether to begin initial treatment with surgery or neoadjuvant chemotherapy. Informed consent was obtained from each patient for treatment.

Paraffin sections of human ovarian epithelial adenocarcinoma tissue samples were obtained from 193 patients who underwent the main part of the series of initial treatments that included surgery at our hospital from January 2005 to December 2011. We studied all cases for which we could obtain both clinical information and paraffin sections. The last specimens taken during the series of initial treatments in one case with invasive carcinoma lesions were used for evaluation of spectrin *α*II expression. Biopsy specimens and recurrent specimens were omitted from evaluation of spectrin *α*II expression. The histological type was assigned according to criteria of the World Health Organization classification (2003) 13. Clinical staging was reviewed based on staging criteria of the International Federation of Gynecology and Obstetrics 1988 (FIGO) 14. FIGO Stage I, II, III, and IV cases were studied.

### Patient follow‐up

The clinical information was obtained from the case records of the 193 patients. Data recorded up to the last date of follow‐up in December 2014 were compiled. Overall survival was defined as the time elapsed between the date of the last surgery in the series of initial treatments and the date of last follow‐up or the date of death. Progression‐free survival was defined as the time elapsed between the date of the last surgery in the series of initial treatments and the date of last follow‐up or the date of progression or recurrence. Progression and recurrence were defined as detection of a new or progressive lesion by image diagnosis with CT (computer tomography), MRI (magnetic resonance imaging), or PET‐CT (positron emission tomography‐computer tomography). Progressive disease was defined according to the revised RECIST (response evaluation criteria in solid tumors) guideline (version 1.1, Kingston, ON, Canada) 15. Survival after recurrence was defined as the time elapsed from the date of progression or recurrence and the date of death or the date of last follow‐up. Overall survival, progression‐free survival, and survival after recurrence are shown for a maximum of 5 years.

### Immunohistochemistry

Tissue sections were cut to a thickness of 3 *μ*m. Immunohistochemical staining was performed with Ventana *i*VIEW DAB Universal Kit using the Ventana XT system BenchMark^®^ (Roche Diagnostics Co. Ltd., Tokyo, Japan). Heat‐induced epitope retrieval was performed by heating deparaffinized sections in citrate buffer (0.01 mol/L, pH 6.0) for 15 min at 95°C. The anti‐spectrin *α*II antibody SPTAN1/Alpha II‐spectrin IHC Antibody (Bethyl Laboratory, Inc., Montgomery, TX) was used at a dilution of 1:100. The slides were developed with 3, 3′‐Diaminobenzidine and were counterstained with methylene blue.

### Evaluation of staining

Spectrin *α*II expression was scored according to the scoring criteria used in HER2 (human epidermal growth factor receptor 2) testing of breast cancer: positive 3+, equivocal 2+, and negative 0 or 1+ 16, 17. The results were assessed independently by observers who did not know any details regarding patient background. In this study, patients were grouped as either positive or non‐positive for spectrin *α*II expression. Patients in the positive group scored 3+, which clearly indicate strong spectrin *α*II expression. Patients in the non‐positive group scored 0, 1+, or 2+.

### Statistical analysis

The clinical variables except spectrin *α*II expression were known before the start of postoperation chemotherapy. If spectrin *α*II expression is routinely tested for, this is another useful clinical variable we will be able to use as a predictor of prognosis before postsurgery chemotherapy is administered. The association between histological types and spectrin *α*II expression scores, and the association between positive versus non‐positive spectrin *α*II expression and clinical parameters were evaluated by the *χ* ^2^‐test. Survival curves were estimated by the Kaplan–Meier method, and compared and analyzed by the log‐rank test. The prognostic significance of spectrin *α*II expression in relation to other clinical variables was assessed by the univariate and multivariate Cox's proportional hazards models. All statistical analyses were performed with Excel Statistics (version 1.00) software (Social Survey Research Information Co., Ltd., Tokyo, Japan). A *P*‐value of <0.05 was considered significant.

### Consent

Written informed consent was obtained from each patient individually for the ovarian cancer anticancer drug resistance study. This study was reviewed and approved by the Ethical Committee, registration number 756.

## Results

### Immunohistochemical detection of spectrin *α*II in various histological types of ovarian adenocarcinoma

**Figure 1:**
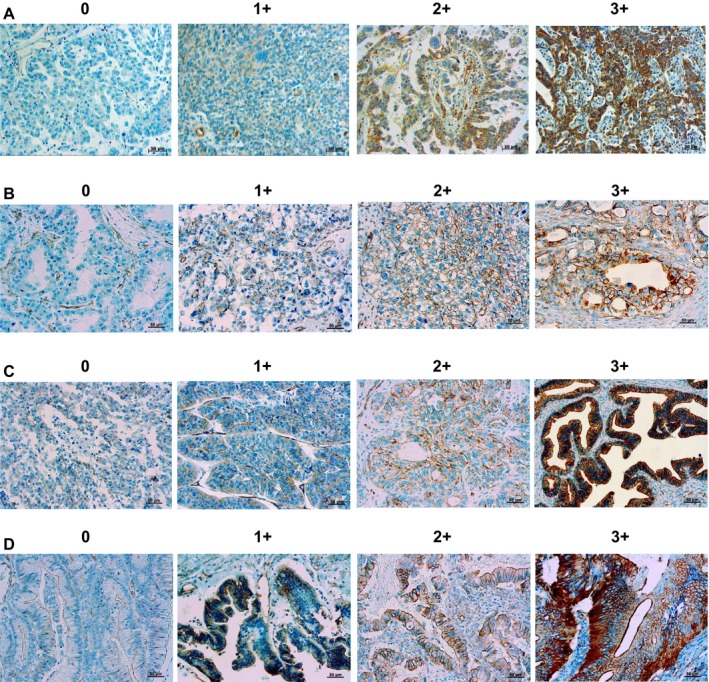
Immunohistochemical staining for spectrin *α*II expression in ovarian epithelial adenocarcinoma. Representative results are shown for serous adenocarcinoma (A), clear cell adenocarcinoma (B), endometrioid adenocarcinoma (C), and mucinous adenocarcinoma (D), and for staining strength negative scores 0 and 1+, equivocal score 2+, and positive score 3+.

Immunohistochemical stained paraffin sections of surgical specimens of ovarian epithelial carcinoma are shown in Figure 1. Serous adenocarcinoma, clear cell adenocarcinoma, endometrioid adenocarcinoma, and mucinous adenocarcinoma are shown in Figure. 1A–D, respectively. Scores 0, 1+, 2+, and 3+ are shown for each histological type. The very clear immunohistochemical staining obtained was of high enough quality for scoring on the cell membrane.

### Histological type, FIGO stage, and spectrin *α*II expression

The distribution of the histological type, FIGO stage, and spectrin *α*II expression of the 193 patients is shown in Table 1. The total number of patients positive for spectrin *α*II expression was 39 (20.2%). Of the 193 cases, 66 (34.2%) were cases of serous adenocarcinoma. Twenty‐two (33.3%) of the 66 serous adenocarcinoma cases scored positive for spectrin *α*II expression. The 61 cases of clear cell adenocarcinoma accounted for 31.6% of the total. The percent of occurrence of this histological type is known to be high in Japan 18. The majority of clear cell adenocarcinoma cases were FIGO stage I (43 cases, 70.5%). Most of the cases scored non‐positive for spectrin *α*II expression. Only four cases (6.6%), all FIGO stage I, scored positive for spectrin *α*II expression. The 38 cases of endometrioid adenocarcinoma accounted for 19.7% of the total. The four cases (10.5%) that scored positive for spectrin *α*II expression were FIGO stage I and II. The 14 cases of mucinous adenocarcinoma accounted for 7.3% of the total. Of these, there was only one advanced case (FIGO stage III). All four cases (28.5%) that scored positive for spectrin *α*II were FIGO stage I. The category “Others” includes other types of adenocarcinoma of four histological subtype. Of these 14 cases, five (35.7%) scored positive for spectrin *α*II and four of the five cases were in the advanced stage. There was an evident relation between spectrin *α*II staining score (0, 1+, 2+, 3+) and histology (*P *< 0.001 *χ* ^2^‐test using Yate's correction). The multiple comparisons of the 10 pairs of five histological types were performed using the Bonferroni correction. Of the 193 cases analyzed, we found a significant difference between serous and clear cell adenocarcinoma (*P *< 0.001) and between clear cell adenocarcinoma (*P *= 0.03). Serous and “others” had higher proportion of spectrin *α*II positivity. There was no significant difference in the other eight pairs of histological types.

**Table 1:** 193 cases of ovarian adenocarcinoma classified according to histological type, FIGO stage, and spectrin *α*II expression

FIGO stage	Spectrin *α*II staining score				
	0	1+	2+	3+	
All histological types					Total (%)
I	28	24	17	14	83 (43.0)
II	7	3	7	2	19 (9.8)
III	14	15	27	20	76 (39.4)
IV	3	2	7	3	15 (7.8)
Total (%)	52 (26.9)	44 (22.8)	58 (30.1)	39 (20.2)	193 (100)
Serous					Total (% in serous)
I	0	1	4	3	8 (12.1)
II	1	0	3	0	4 (6.1)
III	6	7	15	17	45 (68.2)
IV	2	2	3	2	9 (13.6)
Total (% in serous)	9 (13.6)	10 (15.2)	25 (37.9)	22 (33.3)	66 (34.2% in all)
Clear cell					Total (% in Clear cell)
I	20	15	4	4	43 (70.5)
II	2	1	0	0	3 (4.9)
III	5	6	4	0	15 (24.6)
IV	0	0	0	0	0 (0.0)
Total (% in Clear cell)	27 (44.3)	22 (36.1)	8 (13.1)	4 (6.6)	61 (31.6% in all)
Endometrioid					Total (% in endometrioid)
I	4	6	5	3	18 (47.4)
II	3	2	4	1	10 (26.3)
III	2	1	4	0	7 (18.4)
IV	1	0	2	0	3 (7.9)
Total (% in endometrioid)	10 (26.3)	9 (23.7)	15 (39.5)	4 (10.5)	38 (19.7% in all)
Mucious					Total (% in mucinos)
I	3	2	3	4	12 (85.7)
II	1	0	0	0	1 (7.1)
III	0	0	1	0	1 (7.1)
IV	0	0	0	0	0
Total (% in mucinos)	4 (28.6)	2 (14.3)	4 (28.6)	4 (28.6)	14 (7.3% in all)
Others					Total (% in others)
I	1	0	1	0	2 (14.3)
II	0	0	0	1	1 (7.1)
III	1	1	3	3	8 (57.1)
IV	0	0	2	1	3 (21.4)
Total (% in others)	2 (14.3)	1 (7.1)	6 (42.9)	5 (35.7)	14 (7.3% in all)

The category “Others” includes other types of adenocarcinoma of the 4 histological subtypes: mixed type epithelial adenocarcinoma (5 cases), undifferentiated adenocarcinoma (5 cases), and unclassified adenocarcinoma (4 casess).

### Spectrin *α*II expression is associated with survival in 193 cases encompassing all histological types of adenocarcinoma

The 193 patients studied were divided into two groups by the spectrin *α*II expression score: positive (39 patients scoring 3+) and non‐positive (154 patients scoring 0, 1+, and 2+). Spectrin *α*II expression in relation to other clinical parameters is shown in Table 2. The 193 patients were classified by median age; FIGO stage, which was further classified as either early stage (I and II) or advanced stage (III and IV); whether neoadjuvant chemotherapy was administered; whether the patient underwent optimal or suboptimal surgery; and histology. The patients were divided in five groups according to histological type. There was no significant correlation between spectrin *α*II expression and age, FIGO stage, or whether the patient underwent optimal or suboptimal surgery. There was an evident relation between spectrin *α*II expression and neoadjuvant chemotherapy and histology. The multiple comparisons of the 10 pairs of five histological types were performed using the Bonferroni correction. Of the 193 cases analyzed, we found a significant difference between serous and clear cell adenocarcinoma (*P *= 0.0019). There was no significant difference in the other nine pairs of histological types.

**Table 2:** Relationship between spectrin *α*II expression and clinical parameters in 193 cases of ovarian adenocarcinoma

Parameter	Number	Spectrin *α* II Expression		*χ* ^2^ test
		Non‐Positive	Positive	
		Number (%)	Number (%)	
Total	193	154 (79.8)	39 (20.2)	–
Age				
≤55	97	81 (83.5)	16 (16.5)	0.2
≥56	96	73 (76.0)	23 (24.0)	
FIGO stage (Early: stage I and II or advanced: III and IV)				
Early	102	86 (84.3)	16 (15.7)	0.098
Advanced	91	68 (74.7)	23 (25.3)	
Neoadjuvant chemotherapy				
None	147	122 (83.0)	25 (17.0)	0.048^a^
Administered	46	32 (69.6)	14 (30.4)	
Surgery				
Optimal	164	132 (80.5)	32 (19.5)	0.57
Suboptimal	29	22 (75.9)	7 (24.1)	
Histology				
Serous	66	44 (66.7)	22 (33.3)	0.0036^a^,^b^
Clear Cell	61	57 (93.4)	4 (6.6)	
Endometrioid	38	34 (89.5)	4 (10.5)	
Mucinous	14	10 (71.4)	4 (28.6)	
Others	14	9 (64.3)	5 (35.7)	

Multiple comparisons of the 10 pairs of five histological types were performed. There was a significant difference between serous and clear cell adenocarcinoma using the Bonferroni correction (*P *= 0.0019). There was no significant difference in the other nine pairs of histological types.

Non‐Positive: 0, 1+, 2+. Positive: 3+.

*P*‐value shows non‐positive versus positive in each parameter.

a Shows significant.

b Shows Yates' correction.

**Figure 2:**
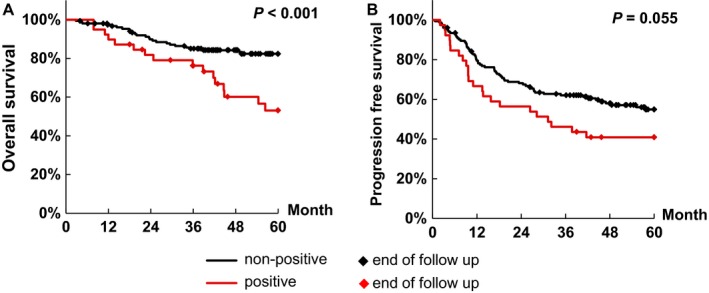
Kaplan–Meier survival curves for patients with ovarian adenocarcinoma according to immunoexpression of spectrin *α*II. (A) Overall survival, and (B) Progression‐free survival. The black line represents non‐positive spectrin *α*II expression and the red line represents positive spectrin *α*II expression. (A) The group positive for spectrin *α*II expression (*N *= 39) had significantly (*P *< 0.001) worse overall survival than the non‐positive group (*N* = 154). (B) There was no significant (*P *= 0.055) difference in either the positive group (*N *= 39) or the non‐positive group (*N *= 153). One case in the non‐positive group was omitted because the patient died with unknown origin at 2.8 months after surgery without recurrence.

We investigated the association of spectrin *α*II expression with overall survival (Table 3). The median patient follow‐up was 53.7 months (range: 2.8–60). The 5‐year survival rate was 76.2% for all 193 patients. The parameters analyzed were spectrin *α*II expression, age, FIGO stage, whether neoadjuvant chemotherapy was administrated, whether the patient underwent optimal or suboptimal surgery, and histology. According to spectrin *α*II expression, the Kaplan–Meier model estimated 5‐year overall survival rates of 82.4% for the non‐positive group and 53.1% for the positive group. Overall survival in the positive group was significantly lower than in the non‐positive group (*P *< 0.001). The Kaplan–Meier survival curve is shown in Figure 2A. Overall survival was significantly different between the two groups when compared by log‐rank test in FIGO stage (*P *< 0.001), neoadjuvant chemotherapy (*P *= 0.0090), and surgery (*P *< 0.001). Overall survival was also significantly different among the five histological types when compared by the log‐rank test (*P *= 0.021). Age and neoadjuvant chemotherapy were not significantly associated with overall survival.

**Table 3:** Overall survival analysis in relation to clinical parameters in 193 of cases ovarian adenocarcinoma

Parameter	Number	Kaplan–Meier analysis		Cox's proportional hazards model							
		5‐year survival (%)	Log‐rank test	Univariate analysis				Multivariate analysis			
			*P*‐value	H. R.	95%	CI	*P*‐value	H. R.	95%	CI	*P*‐value
Total	193	76.2	–	–	–		–	–	–		–
Spectrin *α*II expression											
Non‐positive	154	82.4	0.001>^a^	1				1			
Positive	39	53.1		2.77	1.48	5.20	0.0015^a^	3.77	1.77	8.00	0.001>^a^
Age											
≤55	97	71.5	0.092	1				1			
≥56	96	81.6		0.58	0.31	1.10	0.096	0.77	0.40	1.50	0.44
FIGO stage (Early: stage I and II or Advanced: III and IV)											
Early	102	93.7	0.001>^a^	1				1			
Advanced	91	55.3		11.4	4.44	29	0.001>^a^	12.8	4.09	40.4	0.001>^a^
Neoadjuvant chemotherapy											
None	147	81.2	0.0090^a^	1				1			
Administrated	46	59.6		2.26	1.21	4.24	0.011^a^	1.20	0.57	2.55	0.63
Surgery											
Optimal	164	84.0	0.001>^a^	1				1			
Suboptimal	29	30.1		8.01	4.92	15	0.001>^a^	3.87	1.88	7.98	0.001>^a^
Histology											
Serous	66	65.3	0.021^a^	1				1			
Clear Cell	61	80.4		0.54	0.26	1.13	0.10	4.97	1.83	13.5	0.0016^a^
Endometrioid	38	87.9		0.29	0.10	0.86	0.026^a^	2.01	0.59	6.89	0.27
Mucinous	14	92.3		0.21	0.028	1.58	0.13	2.97	0.31	27.8	0.34
Others	14	59.4		1.49	0.56	3.97	0.43	1.80	0.66	4.92	0.25

Non‐positive: 0, 1+, 2+. Positive: 3+.

HR, hazard ratio; 95% CI, 95% confidence interval.

a Shows significant.

Overall survival in the positive group was significantly lower than in the non‐positive group by both univariate analysis (Hazard ratio [HR]: 2.77, 95% confidence interval [CI]: 1.48–5.20, *P *= 0.0015) and in multivariate analysis (HR: 3.77, 95% CI: 1.77–8.00, *P *< 0.001). These data identify spectrin *α*II expression as an independent predictive factor. FIGO stage, whether the patient underwent optimal or suboptimal surgery, and clear cell adenocarcinoma were also identified as independent predictive factors for overall survival. Age and neoadjuvant chemotherapy were not identified as independent predictive factors.

Next, we investigated progression‐free survival. One case in the non‐positive group was omitted because the patient died with unknown origin at 2.8 months after surgery without recurrence. The remaining 192 patients were divided into positive (*N *= 39) and non‐positive (*N *= 153) groups by spectrin *α*II expression. The Kaplan–Meier survival curve is shown in Figure 2B. There was no significant difference between the positive group and the non‐positive group (*P *= 0.055). Spectrin *α*II expression was not identified as an independent predictive factor by Cox's proportional hazards multivariate analysis (HR: 1.39, 95% CI: 0.84–2.33, *P *= 0.20).

Because we found no significant association of spectrin *α*II expression with progression‐free survival, we investigated survival after recurrence of 92 recurrent cases. The 92 patients studied were divided into two groups by spectrin *α*II expression score: non‐positive (*N *= 69) and positive (*N *= 23). Spectrin *α*II expression in relation to clinical parameters is shown in Table 4. The 92 cases were categorized according to the same parameters as the 193 cases. There was no significant correlation between spectrin *α*II expression and age, FIGO stage, whether neoadjuvant chemotherapy was administrated, surgery, or histology. The multiple comparisons of the 10 pairs of five histological types were performed using the Bonferroni correction. Of the 92 recurrent cases, there was no significant difference between any of the 10 pairs of five tissue types.

**Table 4:** Relationship between Spectrin *α*II expression and clinical parameters in 92 cases of recurred ovarian adenocarcinoma

Parameter	Number	Spectrin *α* II Expression		*χ* ^2^ test
		Non‐Positive	Positive	
		Number (%)	Number (%)	
Total	92	69 (75.0)	23 (25.0)	–
Age				
≤55	52	41 (78.8)	11 (21.2)	0.33
≥56	40	28 (70.0)	12 (30.0)	
FIGO stage (Early: stage I and II or Advanced: III and IV)				
Early	19	16 (84.2)	3 (15.8)	0.46^a^
Advanced	73	53 (72.6)	20 (27.4)	
Neoadjuvant Chemotherapy				
None	52	41 (78.8)	11 (21.2)	0.33
Administered	40	28 (70.0)	12 (30.0)	
Surgery				
Optimal	66	49 (74.2)	17 (25.8)	0.79
Suboptimal	26	20 (76.9)	6 (23.1)	
Histology				
Serous	49	30 (63.8)	17 (36.4)	0.22^a^
Clear cell	18	17 (94.4)	1 (5.6)	
Endometrioid	11	10 (90.9)	1 (9.1)	
Mucinous	3	3 (100)	0 (0.0)	
Others	11	7 (63.6)	4 (36.4)	

Non‐Positive: 0, 1+, 2+: Positive: 3+.

*P*‐value shows non‐positive versus positive in each parameter. Multiple comparisons of the 10 pairs of 5 histological types were performed using the Bonferroni correction. There was no significant difference between any two pairs of histological types.

a Shows Yates' correction.

**Figure 3:**
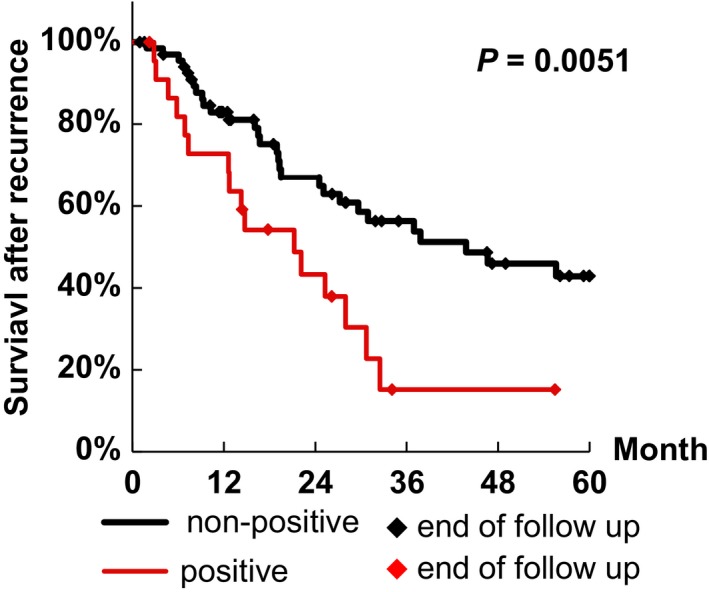
Kaplan–Meier survival curves for patients with recurrent ovarian adenocarcinoma according to immunoexpression of spectrin *α*II. The black line represents non‐positive spectrin *α*II expression and the red line represents positive spectrin *α*II expression. The group positive for spectrin *α*II expression (*N *= 23) had significantly (*P *= 0.0051) worse survival after recurrence than the non‐positive group (*N *= 69).

Survival analyses after recurrence were shown in Table 5. The Kaplan–Meier model estimated a 3‐year survival ratio of 46.0% for all 92 recurred cases. By spectrin *α*II expression, the 3‐year survival rates after recurrence were 56.3% for the non‐positive group and 15.2% for the positive group. Survival after recurrence in the positive group was significantly lower than in the non‐positive group (*P *= 0.0051). The Kaplan–Meier survival curve is shown in Figure 3. Age, FIGO stage, and neoadjuvant chemotherapy were not significantly associated with survival after recurrence. Surgery was also associated with survival after recurrence. Survival after recurrence was significantly different among the five histological types when compared by the log‐rank test. No patients with recurred clear cell adenocarcinoma survived 3 years.

**Table 5:** Survival analysis after recurrence in relation to clinical parameters, in 92 recurred ovarian adenocarcinoma

Parameter	Number	Kaplan–Meier analysis		Cox's proportional hazards model							
		3‐year survival (%)	Log‐rank test	Univariate analysis				Multivariate analysis			
			*P*‐value	H. R.	95%	CI	*P*‐value	H. R.	95%	CI	*P*‐value
Total	92	46.0	–	–	–		–	–	–		–
Spectrin *α* II expression											
Non‐positive	69	56.3	0.0051^a^	1				1			
Positive	23	15.2		2.39	1.27	4.47	0.0066^a^	4.49	2.06	9.79	0.001>^a^
Age											
≤55	52	42.8	0.38	1		1.41	0.38	1			
≥56	40	51.3		0.76	0.41			0.91	0.48	1.75	0.78
FIGO stage (Early: stage I and II or advanced: III and IV)											
Early	19	63.7	0.30	1		3.45	0.30	1			
Advanced	73	42.2		1.53	0.67			1.50	0.51	4.39	0.46
Neoadjuvant chemotherapy											
None	52	42.7	0.22	1				1			
Administrated	40	50.8		0.69	0.38	1.25	0.22	1.04	0.46	2.34	0.92
Surgery											
Optimal	66	53.2	0.0067^a^	1				1			
Suboptimal	26	28.5		2.27	1.23	4.18	0.0084^a^	2.36	1.04	5.39	0.041^a^
Histology											
Serous	49	55.2	0.0020^a^	1				1			
Clear cell	18	0.0		4.13	1.94	8.79	0.001>^a^	8.88	3.44	22.9	0.001>^a^
Endometrioid	11	60.6		1.00	0.40	2.48	0.99	2.20	0.78	6.22	0.14
Mucinous	3	50.0		0.87	0.12	6.61	0.91	2.87	0.32	26. 1	0.35
Others	11	36.6		1.62	0.61	4.32	0.34	1.39	0.50	3.83	0.53

Non‐positive: 0, 1+, 2+. Positive: 3+.

HR, hazard ratio; 95% CI, 95% confidence interval.

a shows significant.

Survival after recurrence in the group positive for spectrin *α*II expression was significantly lower than in the non‐positive group by both univariate analysis (HR: 2.39, 95% CI: 1.27–4.47, *P *= 0.0066) and multivariate analysis (HR: 4.49, 95% CI: 2.06–9.79, *P *< 0.001). These data identified spectrin *α*II expression as an independent predictive factor for survival after recurrence. Age, FIGO stage, and neoadjuvant chemotherapy, were not identified as independent predictive factors by multivariate analysis. Surgery and clear cell adenocarcinoma in the histology category were also identified as independent predictive factors for survival after recurrence.

### Spectrin *α*II expression is associated with overall survival in 66 cases of serous adenocarcinoma and in 127 cases of non‐serous adenocarcinoma

We previously found spectrin *α*II expressed in serous adenocarcinoma cells, so we concentrated on serous adenocarcinoma patients in a detailed partial study. The 66 patients studied were divided into two groups by the spectrin *α*II expression score: non‐positive (44 patients) and positive (22 patients). The 66 cases were categorized according to the same clinical parameters as the 193 cases. The association of spectrin *α*II expression with overall survival was analyzed (Table 6). The median patient follow‐up was 46.5 months (range: 2.8–60). According to spectrin *α*II expression, the Kaplan–Meier model estimated 5‐year overall survival rates of 77.5% for the non‐positive group and 40.4% for the positive group. Overall survival for the positive group was significantly lower than for the non‐positive group (*P *= 0.0093). Overall survival for the group positive for spectrin *α*II expression was significantly lower than for the non‐positive group by both univariate analysis (HR: 3.05; 95% CI: 1.26–7.39; *P *= 0.013) and multivariate analysis (HR: 3.33; 95% CI, 1.33–8.45; *P *= 0.010). Thus, spectrin *α*II expression was identified by the data as independent predictive factors of overall survival in serous adenocarcinoma.

**Table 6:** Analyses of overall survival in relation to clinical parameters in 66 serous adenocarcinoma cases

Parameter	Number	Kaplan–Meier Model		Cox's proportional hazards model							
		5‐year survival (%)	Log‐rank Test	Univariate analysis				Multivariate analysis			
			*P*‐value	HR	95%	CI	*P*‐value	HR	95%	CI	*P*‐value
Total	66	64.0	–	–	–	–	–	–	–	–	–
Spectrin *α* II Expression											
Non‐positive	44	77.5	0.0093^a^	1				1			
Positive	22	40.4		3.05	1.26	7.39	0.013^a^	3.33	1.33	8.45	0.010^a^
Age											
≤57	33	64.7	0.90	1				1			
≥58	33	66.8		0.95	0.39	2.29	0.90	1.93	0.71	5.22	0.20
FIGO Stage (Early: stage I and II or advanced: III and IV)											
Early	12	90	0.051	1				1			
Advanced	54	59.4		5.84	0.78	43.7	0.086	3.01	0.35	26.1	0.32
Neoadjuvant chemotherapy											
None	33	72.9	0.27	1				1			
Administered	33	56.7		1.65	0.68	4.05	0.27	1.70	0.65	4.46	0.28
Surgery											
Optimal	49	76.2	0.001>^a^	1				1			
Suboptimal	17	34.8		4.09	1.70	9.88	0.0017^a^	4.37	1.63	11.7	0.0034^a^

Non‐positive: 0, 1+, 2+. Positive: 3+.

HR, hazard ratio; 95% CI, 95% confidence interval.

There was no significant correlation between spectrin *α*II expression and other four parameters (age: *P *= 1.00, International Federation of Gynecology and Obstetrics (FIGO) stage: *P *= 0.73, neoadjuvant chemotherapy: *P *= 1.00, and surgery: *P *= 0.84).

a Shows significant.

Next, we investigated the overall survival of 127 cases of non‐serous adenocarcinoma. We classified clear cell, endometrioid, mucinous, and other adenocarcinomas as non‐serous adenocarcinoma. The 127 patients were divided into two groups by the spectrin *α*II expression score: non‐positive (*N *= 110) and positive (*N *= 17). The 127 cases were categorized according to the same clinical parameters as the 193 cases. Overall survival was statistically analyzed (Table 7). The median patient follow‐up was 56.4 months (range: 4–60). The data identified spectrin *α*II expression as an independent predictive factor for overall survival of non‐serous adenocarcinoma patients by multivariate analysis (HR: 6.39, 95% CI: 1.97–20.8, *P *= 0.0020).

**Table 7:** Analyses of overall survival in relation to clinical parameters in 127 non‐serous adenocarcinoma cases

Parameter	Number	Kaplan–Meier Model		Cox's proportional hazards model							
		5‐year survival (%)	Log‐rank Test	Univariate analysis				Multivariate analysis			
			*P*‐value	H. R.	95%	CI	*P*‐value	H. R.	95%	CI	*P*‐value
Total	127	81.7	–	–	–	–	–	–	–	–	–
Spectrin *α* II Expression											
Non‐positive	110	84.3	0.18	1				1			
Positive	17	67.2		1.97	0.72	5.28	0.19	6.39	1.97	20.8	0.0020^a^
Age											
≤55	68	76.7	0.085	1				1			
≥56	59	87.8		0.45	0.17	1.15	0.094	0.47	0.18	1.25	0.13
FIGO stage (Early: stage I and II or advanced: III and IV)											
Early	90	94.2	0.001>^a^	1				1			
Advanced	37	49.3		15.7	5.24	47	0.001>^a^	18.6	50.0	69.3	0.001>^a^
Neoadjuvant chemotherapy											
None	114	83.4	0.16	1				1			
Administered	13	64.3		2.14	0.72	6.38	0.17	0.36	0.10	1.21	0.099
Surgery											
Optimal	115	87.2	0.001>^a^	1				1			
Suboptimal	12	21.9		15.5	6.20	38.9	0.001>^a^	4.36	1.57	12.1	0.0047^a^

Non‐positive: 0, 1+, 2+. Positive: 3+.

HR,hazard ratio; 95% CI, 95% confidence interval.

There was no significant correlation between spectrin *α*II expression and age (*P *= 0.27), International Federation of Gynecology and Obstetrics (FIGO) stage (*P *= 0.80), neoadjuvant chemotherapy (*P *= 0.51), or surgery (*P *= 0.92).

a Shows significant.

## Discussion

This study demonstrates that spectrin *α*II expression is an independent predictive factor for overall survival in ovarian adenocarcinoma. Because spectrin *α*II expression was not significantly associated with progression‐free survival, recurrent cases were studied. Our study indicates that spectrin *α*II expression is an independent predictive factor for survival after recurrence in ovarian adenocarcinoma. We considered that prognoses of patients in this study were approximate to their sensitivity to anticancer drug. Although prognoses were affected by tumor activity, locations of invasion, metastasis, and other many factors, we considered that anticancer drug sensitivity was the most important factor in the prognosis of the group of patients that received aggressive chemotherapy treatment.

Fortunately, the new anti‐spectrin *α*II antibody used in this study produced much better quality immunohistochemical staining of clinical specimens than the anti‐spectrin *β*II antibody used in the previous study. The strong and specific staining produced was sufficient to evaluate the staining strength according to the guideline for HER2 testing of breast cancer. It was also fortunate that HER2 testing of breast cancer could be applied to evaluation of spectrin *α*II expression. The next step will be to evaluate spectrin *α*II gene expression by FISH test, which is used in testing for the HER2 gene.

The prongoses for patients scoring 3+ were remarkably different compared with the prongoses for patients scoring 0, 1+, and 2+, which is why the patients were divided into two groups. Overall survival was compared and analyzed by the log‐rank tests. There was no significant difference between negative (score 0, 1+) and equivocal (score 2+) groups (*P *= 0.64). However, overall survival of the positive group (score 3+) was significantly lower than either negative (*P *= 0.038) or equivocal (*P *= 0.023) group.

At the beginning of this study, spectrin *α*II was considered to be a more important factor in the prognosis of serous adenocarcinoma than of other histological types because spectrin *α*II and *β*II proteins were discovered in cisplatin‐resistant ovarian serous adenocarcinoma cells in the previous study. The biological mechanism of drug resistance is that spectrin *α*II‐*β*II tetramers bind to glutathione–platinum complexes to produce spectrin–glutathione–platinum complexes. Spectrin *α*II‐*β*II tetramers are cytoskeletons supporting the cell membrane, and anchor glutathione–platinum complexes. The existence of spectrin *α*II arrests cisplatin or platinum drug activity. Because this theory is not affected by histological types, we consider that spectrin *α*II functions similarly in non‐serous adenocarcinoma. In this study, approximately 20% of the tissue specimens of all histological types scored positive for spectrin *α*II expression, so it was appropriate to investigate not only serous adenocarcinoma but also non‐serous adenocarcinoma. Therefore, the study considered whether spectrin *α*II expression could be used to predict overall survival of whole histological type of adenocarcinoma patients. As expected, Spectrin *α*II expression was identified as an independent predictive factor for overall survival ranking in importance with the variables of FIGO stage, whether a patient underwent optimal or suboptimal surgery, and clear cell adenocarcinoma.

Spectrin *α*II expression was not an independent factor of progression‐free survival. On the other hand, FIGO stage, neoadjuvant chemotherapy, and surgery were independent factors in multivariate analysis of progression‐free survival. Previously, spectrin *α*II was purified from cisplatin‐resistant cells and shown to contribute to platinum anticancer drug resistance. We considered that progression‐free survival was affected by these three parameters more than by anticancer drug sensitivity related to spectrin *α*II expression. The three parameters concern the remnant volume at the beginning of the day count, which is the day of the last surgery in the series of initial treatments. We think that the larger tumor remnant, the faster it would grow and could be detected earlier by image diagnosis. Although anticancer drug sensitivity is an important factor for ovarian cancer treatment, the effect is masked by the three parameters after the last surgery in the series of initial treatments.

The difference between overall survival and progression‐free survival was examined. The period of survival after recurrence for 92 recurred cases was calculated by subtracting the progression‐free survival period from the overall survival period. Spectrin *α*II expression was also found to be an independent predictive factor for survival after recurrence. Recurrent patients are mainly treated with chemotherapy and surgical options are limited. Identification of spectrin *α*II expression as an independent predictive factor for survival after recurrence indicates the association of spectrin *α*II expression with chemosensitivity of recurred tumors large enough to be detected by image diagnosis.

In the Histology category in Table 3, hazard ratio values showed opposite directions in univariate analysis and multivariate analysis. Because clear cell, endometrioid, and mucinous adenocarcinoma groups included more early stage cases than the serous adenocarcinoma group (Table 1), the prognoses for these three groups were better than for the serous adenocarcinoma group according to the Histology parameter. Hazard ratio values for the clear cell, endometrioid, and mucinous adenocarcinoma groups were primarily affected by FIGO stage, secondly by surgery, and thirdly by spectrin *α*II expression. The primary aim of this study is to show that spectrin *α*II expression is a prognostic factor. Although the relation between histology and spectrin *α*II expression is important, we consider that it can safely be said that spectrin *α*II expression is one of the prognostic factors.

It is necessary to consider several biases because this is a retrospective study. A comparison of statistics in this study with Japanese statistics of ovarian cancer, gives prognoses that are better than the average in Japan. The 5‐year survival rate of this study was 76.2% in contrast to the average in Japan of 70.2%. No data is available on rates of positive spectrin *α*II expression in the average statistics for Japan. However, we believe it is very unlikely that our data on positive spectrin *α*II expression rates would be lower in comparison if such data did exist. We also believe it is very unlikely that the good prognoses reported in this study are due to the positive spectrin *α*II expression rates we obtained. Furthermore, factors such as tumor grade should be considered as biases in evaluating staining strength. We think that tumor cellularity may affect staining strength. Immunological staining was performed with the Ventana XT system, and spectrin *α*II expression was scored according to the score criteria in HER2 testing of breast cancer. HER2 testing is well established and routinely performed. We made an effort to minimize bias by having the evaluation of spectrin *α*II expression performed by experienced pathologists and trained medical doctors. We believe that this lends reliability to the study. Analysis of greater numbers of tissue samples will enable us to formulate better staining standards including tumor grade evaluation.

This study design was appropriate for investigating platinum drug resistance, primarily resistance to carboplatin, in clinical practice because ovarian adenocarcinoma patients treated with the standard combination chemotherapy regimen of paclitaxel and carboplatin were the subject of this study. During the series of initial treatments, carboplatin (cisplatin, in a few cases) was administered to all patients requiring chemotherapy. Only 14 patients in this study did not undergo chemotherapy. Because the number of patients treated with surgery alone was small and the prognosis was good in each case, the 14 patients were considered to have a negligible effect on the analyses. Furthermore, patients treated with bevacizumab are not included in the series of initial treatments, which were administered before this drug was introduced in Japan.

Gynecologic oncologists have experience to treat patients who show both sensitivity and resistance to anticancer drugs. However, there is currently no criterion for determining in advance which patients will be sensitive or resistant to the anticancer drugs commonly administered. Thus, it is important to discover factors associated with anticancer drug sensitivity and prognosis. The findings of this study are a valuable indicator of the potential of spectrin *α*II expression as a useful predictor of anticancer drug sensitivity. However, further research is required. Furthermore, therapeutic methods must be developed to effectively treat patients with carcinoma cells expressing spectrin *α*II. Hopefully, new combinations of anticancer drugs that overcome anticancer drug resistance associated with spectrin *α*II expression will be discovered so that overall survival of advanced‐stage patients who undergo suboptimal surgery and whose ovarian carcinoma cells express spectrin *α*II can be extended. It is hoped that this study will serve as a foundation for developing clinical treatments that successfully overcome anticancer drug resistance in ovarian cancer.

## Conclusions

Spectrin *α*II expression is a useful predictor of anticancer drug resistance and postoperative prognosis in both serous adenocarcinoma and nonserous adenocarcinoma. This study will serve as a foundation for developing clinical treatments that successfully overcome anticancer drug resistance in ovarian cancer.

## Conflict of Interests

None declared.
